# A High-Quality Reference Genome for a Parasitic Bivalve with Doubly Uniparental Inheritance (Bivalvia: Unionida)

**DOI:** 10.1093/gbe/evab029

**Published:** 2021-02-11

**Authors:** Chase H Smith

**Affiliations:** 1 Department of Integrative Biology, University of Texas, Austin, Texas, USA; 2 Biology Department, Baylor University, Waco, Texas, USA

**Keywords:** Pacific Biosciences, 10XGenomics, freshwater mussel, phylogenomics, *Potamilus*

## Abstract

From a genomics perspective, bivalves (Mollusca: Bivalvia) have been poorly explored with the exception for those of high economic value. The bivalve order Unionida, or freshwater mussels, has been of interest in recent genomic studies due to their unique mitochondrial biology and peculiar life cycle. However, genomic studies have been hindered by the lack of a high-quality reference genome. Here, I present a genome assembly of *Potamilus streckersoni* using Pacific Bioscience single-molecule real-time long reads and 10X Genomics-linked read sequencing. Further, I use RNA sequencing from multiple tissue types and life stages to annotate the reference genome. The final assembly was far superior to any previously published freshwater mussel genome and was represented by 2,368 scaffolds (2,472 contigs) and 1,776,755,624 bp, with a scaffold N50 of 2,051,244 bp. A high proportion of the assembly was comprised of repetitive elements (51.03%), aligning with genomic characteristics of other bivalves. The functional annotation returned 52,407 gene models (41,065 protein, 11,342 tRNAs), which was concordant with the estimated number of genes in other freshwater mussel species. This genetic resource, along with future studies developing high-quality genome assemblies and annotations, will be integral toward unraveling the genomic bases of ecologically and evolutionarily important traits in this hyper-diverse group.

**Significance**

The global decline of freshwater mussels has emphasized the need to better understand the biology, ecology, and evolution of the group. The basic genetics of freshwater mussels remain poorly understood despite a recent push to understand factors that contribute to their demise. Recent investigations in freshwater mussels to determine the genomic mechanisms involved with ecologically important traits such their life cycle and sex determination have been hindered by the lack of a high-quality reference genome. Here, I develop a high-quality reference genome for the freshwater mussel species *Potamilus streckersoni* or Brazos Heelsplitter. This genome assembly will facilitate future genome wide association studies to better understand these highly imperiled organisms.

## Introduction

From a genomics perspective, bivalves (Mollusca: Bivalvia) have been poorly explored with the exception for those of high economic value (Liu et al. 2021). One bivalve order of considerable interest is Unionida or freshwater mussels, which consists of over 800 species (Graf and Cummings 2007). Freshwater mussels have a unique mitochondrial (mt) biology: they deviate from strictly maternal inheritance of mt and have a unique mode of mt inheritance called doubly uniparental inheritance (DUI) (e.g., Breton et al. 2007, 2018; Zouros 2013). They also display a peculiar life history that involves a parasitic larval stage (glochidia) that must attach to vertebrate hosts (primarily fish) to complete metamorphosis (Barnhart et al. 2008). While numerous genomic studies have sought to identify genes associated with inherent biological characteristics of mussels (e.g., Luo et al. 2014; Shi et al. 2015; Patnaik et al. 2016; Bertucci et al. 2017; Renaut et al. 2018; Capt et al. 2019), these studies have been hindered by the lack of a high-quality reference genome. Here, I present a genome assembly for the freshwater mussel species *Potamilus streckersoni*, or Brazos Heelsplitter, using Pacific Bioscience single-molecule real-time (PacBio; Menlo Park, CA) long reads and 10X Genomics-linked read sequencing (10X; San Francisco, CA). Further, I use RNA sequencing (RNA-seq) from multiple tissue types and life stages to provide a functional annotation for the assembly. This study will be integral toward unraveling the basic biology of freshwater mussels and the genomic bases of evolutionarily important traits contributing to their diversification.

## Materials and Methods

### Data Generation

One gravid female *P. streckersoni* was collected from the type locality and deposited at Florida Museum (UF439535). Whole genomic DNA was extracted from fresh mantle tissue using the Qiagen PureGene Kit (Hilden, Germany) with standard protocols. High-molecular weight DNA was ensured by visualizing the isolation on a 1% agarose gel stained with GelRed nucleic acid stain (Biotium, Hayward, CA). Isolation quantity and quality was assessed using a Qubit^TM^ fluorometer and a NanoDrop^TM^ One (ThermoFisher Scientific; Waltham, MA), respectively. Before PacBio and 10X sequencing, the initial identification based on external shell morphology was confirmed by amplifying and sequencing the mt gene *NADH dehydrogenase subunit 1* as described in Serb et al. (2003). A PacBio library was size selected with 10 kb cut-off following the SMRT bell construction protocol. The library was sequenced on a single-molecule real-time cell of a PacBio Sequel II using the v 2.0 chemistry. For 10X sequencing, a Chromium 10X library was constructed from high-molecular-weight DNA according to manufacturers recommended protocols. The resulting library was quantitated by qPCR and sequenced on an Illumina NovaSeq 6000 (San Diego, CA).

Total RNA was extracted from five different tissue types from the adult female: foot, gill, gonad, mantle, and stomach. In addition to total RNA extraction of adult somatic and gonadal tissue, RNA was isolated from a pool of fully developed glochidia to pick up additional transcripts expressed at the larval stage. All RNA was extracted using QIAshredder and RNeasy kits as described by the manufacturer (Qiagen). RNA quality and integrity were determined using a NanoDrop^TM^ and an Agilent Bioanalyzer (Santa Clara, CA), respectively. Messenger RNA (mRNA) was purified from approximately 200 ng of total RNA and a mRNA library was prepared using KAPA mRNA HyperPrep Kits (Roche; Basel, Switzerland). Indexed libraries that met appropriate cut-offs were quantified by qRT-PCR and sequenced using 100 bp paired-end sequencing on an Illumina NovaSeq 6000.

### Genome Assembly

A female mitogenome for *P. streckersoni* was de novo assembled with 10X reads using MitoZ v 2.4 (Meng et al. 2019). After assembly, the mitogenome was annotated using MITOS (Bernt et al. 2013) and final limits of transfer RNAs (tRNAs) were assessed using ARWEN (Laslett and Canback 2008).

Before de novo assembly of the nuclear genome, mt reads were removed from 10X and PacBio data using BWA-MEM (Li 2013), minimap2 v 2.17-r941 (Li 2018), and SAMtools v 1.9 (Li et al. 2009). Overall characteristics of the genome (e.g., genome size, repetitive elements) were estimated using 10X data by *k*-mer spectrum distribution analysis for *k *=* *21 in JELLYFISH v 2.2.10 (Marçais and Kingsford 2011) and GENOMESCOPE v 2.0 (Ranallo-Benavidez et al. 2020).

A draft genome assembly was derived from 10X reads using Supernova v 2.1.1 with default parameters (Weisenfeld et al. 2017). For PacBio reads, wtdbg2 v 2.5 (Ruan and Li 2019) was used to assemble a draft genome using the commands “-x rsII -AS 4 -p 19 –tidy-reads 5000 –edge-min 4 –rescue-low-cov-edges” to account for high error rate and coverage. The PacBio assembly had superior contiguity and rather than use the 10X assembly, a pipeline was developed using linked reads to correct and scaffold the PacBio assembly similar to previous studies (e.g., Li et al. 2019; Wallberg et al. 2019).

The wtdbg2 assembly was polished using two iterations of arrow in GCpp v 1.9.0 (Pacific Biosciences 2019) and NextPolish v 1.2.3 (Hu et al. 2020), per developers recommendation for accuracy. The Tigmint+ARKS pipeline in ARCS v 1.1.0 (Coombe et al. 2018; Jackman et al. 2018; Yeo et al. 2018) was used to correct assembly errors and scaffold contigs using 10X data. Default parameters were used except for the barcode read frequency range (-m 20–20,000). LINKS v1.8.7 (Warren et al. 2015) was used to process ARKS results and construct scaffolds using the default parameters except for the ratio of barcode links between two most supported graph edges (-a 0.9). Gaps were introduced within scaffolds due to joins made by ARKS, and TGS-GapCloser v 1.0.1 (Xu et al. 2020) was used to fill gaps with PacBio reads.

Purge Haplotigs (Roach et al. 2018) was used to remove highly heterozygous haplotypes and trim contigs with overlapping ends. Coverage thresholds of 20, 190, and 195 were used to allow Purge Haplotigs to examine all contigs for suspected haplotigs. The curated assembly was subjected to two iterations of polishing in NextPolish. MegaBLAST v 2.10.0 was used to screen for possible contaminated scaffolds by identifying hits with >98% homology to available prokaryotic genomes from NCBI (https://www.ncbi.nlm.nih.gov). I used QUAST v 5.0.2 (Mikheenko et al. 2018) to generate assembly statistics and BUSCO v 4.0.6 (Seppey et al. 2019) to evaluate the completeness of the assembly using the 954 conserved genes in the Metazoan lineage after each step of the pipeline.

### Genome Annotation

RNA-Seq reads were trimmed using TRIM GALORE! v0.6.4 (www.bioinformatics.babraham.ac.uk/projects/trim_galore/) with default parameters except for minimum read length (35 bp). Data quality was ensured in FastQC v 0.11.9 (www.bioinformatics.babraham.ac.uk/projects/fastqc/). Prior to read mapping, repeats in the *P. streckersoni* curated genome assembly were identified and masked using RepeatModeler v 2.0.1 (Flynn et al. 2020) and RepeatMasker v 4.0.9 (Smit et al. 2015), respectfully. RNA-seq reads were mapped to the masked assembly using HISAT2 v 2.1.0 (Kim et al. 2015), and aligned reads were used to train AUGUSTUS v 3.3.3 (Stanke et al. 2008). To incorporate cDNA evidence, rnaSPAdes (Bushmanova et al. 2019) was used to de novo assemble transcriptomes from RNA samples of adult tissues and pooled glochidia, respectively. Transcripts less than 200 bp were removed and remaining transcripts were aligned to the genome assembly using minimap2. Metazoan reference protein sequences were compiled from the OrthoDB protein database v 10 (Kriventseva et al. 2019), and ProtHint v 2.4.0 (Brůna et al. 2020) was used to generate potential borders between coding and noncoding regions.

Structural and function annotation was performed using the Funannotate pipeline v 1.8.0 (Palmer and Stajich 2017). Consensus gene models were produced by EVidenceModeler v 1.1.1 (EVM; Haas et al. 2008) using protein evidence, transcript evidence, and ab initio predictions from AUGUSTUS, GeneMark-ES v 4.61 (Lomsadze 2005), GlimmerHMM (Majoros et al. 2004), and SNAP (Korf 2004). tRNAscan-SE v 2.0.5 (Chan and Lowe 2019) was used to generate tRNA models. Protein annotations were assessed using BUSCO, CAZy (Cantarel et al. 2009), eggNOG (Huerta-Cepas et al. 2019), MEROPS (Rawlings et al. 2018), Pfam (Finn et al. 2014), and UniProt (The UniProt Consortium 2017) domains by eggNOG-mapper v 2 (Huerta-Cepas et al. 2017), InterProScan v 5.47-82.0 (Jones et al. 2014), and BLASTP v 2.10.0.

### Phylogenomic Analysis

RNA-seq reads were compiled for 19 additional taxa distributed across the subclass Paleoheterodonta from the SRA database (supplementary table S2, Supplementary Material online). The larval transcriptome for *P. streckersoni* depicted a higher duplication rate when compared to the adult transcriptome (supplementary table S1, Supplementary Material online); therefore, the adult transcriptome was used for phylogenomic analyses to minimize impacts of heterozygosity. Dataset generation followed similar methods as Kocot et al. (2011, 2019). Reads were quality trimmed using TRIM GALORE! and transcriptomes were de novo assembled in rnaSPAdes. Nucleotide sequences were translated with TRANSDECODER v 5.5.0 (http://transdecoder.sourceforge.net/) and HAMSTR v 13.2.6 (Ebersberger et al. 2009) was used to generate orthologs. Genes were aligned with MAFFT v 7.471 (Katoh and Standley 2013) and were trimmed using ALISCORE (Misof and Misof 2009) and ALICUT v 2.31 (Kück 2009) to remove ambiguously aligned regions. Gene trees were generated in FASTTREE v 2.1.10 (Price et al. 2010) and PHYLOTREEPRUNER (Kocot et al. 2013) was used to select the best sequence for each taxon. Genes represented by 10 or more taxa were retained and concatenated using FASconCAT-G v 1.04 (Kück and Longo 2014). Phylogenetic reconstruction was performed in IQ-TREE v 2.1.2 (Chernomor et al. 2016; Minh et al. 2020) and ModelFinder (Kalyaanamoorthy et al. 2017) was used to select the partitioning scheme and best amino acid substitution models for the analysis. IQ-TREE analyses conducted 10 repetitions of an initial tree search and 1,000 ultrafast bootstrap replicates (ufBS) for nodal support (Hoang et al. 2018), per developers recommendation for accuracy.

## Results and Discussion

PacBio sequencing generated ∼225 Gb with a subread N50 of 29 kb, representing >100× coverage of the genome assembly. 10X sequencing generated 672.03 million 150-bp paired end reads, roughly ∼48× coverage of the assembled genome. The female-type mitogenome for *P. streckersoni* was recovered as a single contig represented by 16,293 bp (supplementary fig. S1, Supplementary Material online) and consisted of 37 genes similar to mitogenomes derived from other *Potamilus* spp. (Feng et al. 2016; Wen et al. 2017): 13 protein-coding genes, 2 ribosomal RNAs, and 22 tRNAs.

**Fig. 1.**
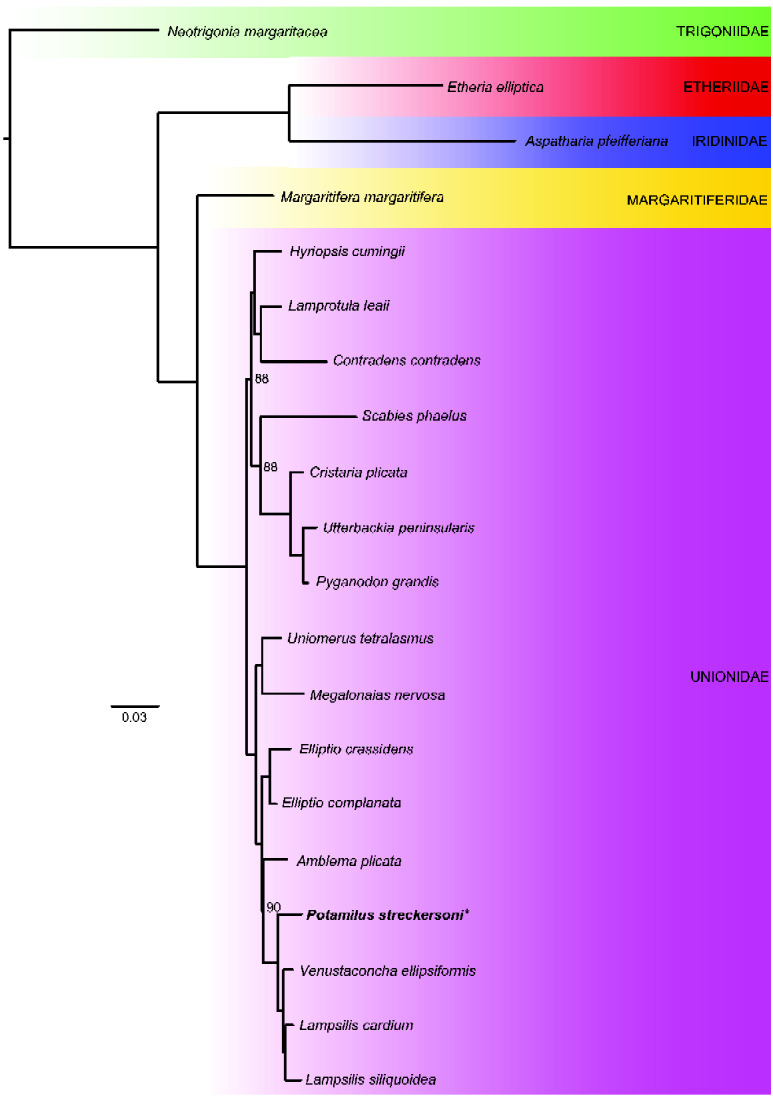
Phylogenetic reconstruction generated from select transcriptomes from subclass Paleoheterodonta and family designations for selected taxa. All but three nodes were represented by full ultrafast bootstrap support.

The final curated genome assembly was represented by 2,368 scaffolds (2,472 contigs), 1,776,755,624 bp, and a scaffold N50 of 2,051,244. Additional information about genome statistics is presented in Table 1. The assembled genome size was concordant with the estimated genome size generated from 10X data (∼1.81 Gb; supplementary fig. S2, Supplementary Material online), and was also similar to the estimated genome size of *Venustaconcha ellipsiformis* (∼1.80 Gb), the most closely related freshwater mussel species with genomic resources (Renaut et al. 2018) (fig. 1; table 2). Overall, 94.6% of the 954 expected metazoan genes were identified in the nuclear genome assembly by BUSCO, which supported the assembly as largely complete. RepeatModeler marked 51.03% of the assembly as repetitive elements (∼0.96 Gb), which was larger than the repetitive element content estimated by JELLYFISH and GENOMESCOPE (32.5%); however, aligns with the percentage of repetitive elements in other bivalve genomes (e.g., Wang et al. 2017; Bai et al. 2019; Gomes-dos-Santos et al. 2020) (table 2). The majority of repetitive elements in the *P. streckersoni* genome assembly were uncharacterized (25.5%; ∼453 Mb) similar to other studies in freshwater mussels (Gomes-dos-Santos et al. 2020), but notable categorizable transposable element compositions were as follows: DNA transposons—13.71% (∼243 Mb), long interspersed nuclear elements—5.01% (∼89 Mb), long terminal repeats—3.09% (∼55 Mb), Penelope-like elements—1.14% (∼20 Mb), and short interspersed nuclear elements—0.01% (∼134 kb).

**Table 1:** QUAST Scaffold Statistics and BUSCO Profiles Based on the Metazoan and mollusca_odb10 Lineage for Each Assembly

Value	PacBio	PacBio + 10X	Final Assembly
Scaffolds	6,770	6,591	2,368
Contigs	6,770	6,877	2,472
Total length	1,847,057,843	1,860,819,131	1,776,755,624
Scaffold N50	1,241,163	1,977,022	2,051,244
Scaffold N75	570,763	915,592	1,056,644
Scaffold L50	436	265	245
Scaffold L75	981	608	546
N’s per 100 kbp	0	0.55	0.53
Complete single copy	892 (93.5%)	891 (93.4%)	894 (93.7%)
Complete duplicated	11 (1.2%)	12 (1.3%)	9 (0.9%)
Fragmented	10 (1.0%)	10 (1.0%)	11 (1.2%)
Missing	41 (4.3%)	41 (4.3%)	40 (4.2%)

Note.—The three steps columns represent the polished assembly derived from wtdbg2 (PacBio), the polished assembly generated from the Tigmint+ARKS pipeline (PacBio + 10X), and the final assembly after removing highly heterozygous haplotypes (final assembly).

**Table 2:** Summary Statistics of Annotated Genome Assemblies Available for Bivalvia

Order	Taxa	Genome Size (Mb)	Scaffold N50 (Kb)	Repeat Content (%)	Number of Genes	Source
Adapedonta	*Sinonovacula constricta*	1,332	57,990	36.65	26,273	Dong et al. (2020)
Arcida	*Scapharca broughtonii*	885	4,500	46.41	24,045	Bai et al. (2019)
Mytilida	*Bathymodiolus platifrons*	1,660	343	47.25	33,584	Sun et al. (2017)
	*Limnoperna fortune*	1,670	312	33.40	60,717	Uliano-Silva et al. (2018)
	*Modiolus philippinarum*	2,630	100	59.66	36,549	Sun et al. (2017)
Ostreida	*Crassostrea gigas*	559	401	34.71	28,072	Zhang et al. (2012)
	*Crassostrea virginica*	685	75,944	39.69	34,596	Gómez-Chiarri et al. (2015)
	*Saccostrea glomerate*	788	804	45.39	29,738	Powell et al. (2018)
Pectinida	*Argopecten purpuratus*	725	1,020	32.04	26,256	Li et al. (2018)
	*Chlamys farreri*	780	602	27.73	28,602	Li et al. (2017)
	*Patinopecten yessoensis*	988	804	27.85	24,738	Wang et al. (2017)
Pteriida	*Pinctada fucata*	1,024	167	43.35	31,447	Takeuchi et al. (2012, 2016)
	*Pinctada fucata martensii*	991	324	48.01	30,815	Du et al. (2017)
Unionida	*Margaritifera margaritifera*	2472	289	59.07	35,119	Gomes-dos-Santos et al. (2020)
	*Megalonaias nervosa*	2,360	53	∼25	49,149	Rogers et al. (2020)
	*Potamilus streckersoni*	1,776	2,051	50.13	41,065	This study
	*Venustaconcha ellipsiformis*	∼1,800	7	36.29	201,068	Renaut et al. (2018)
Venerida	*Archivesica marissinica*	1,520	73,300	42.20	28,949	Ip et al. (2021)
	*Cyclina sinensis*	903	46.5	43.14	27,564	Wei et al. (2020)
	*Ruditapes philippinarum*	1,129	345	38.30	27,652	Yan et al. (2019)

RNA-seq generated ∼13 Gb and transcriptomes based on adult tissue and larvae (96,843 and 104,614 transcripts, respectively) were largely complete based on BUSCO analyses, with each transcriptome having more than 96% of metazoan genes (supplementary table S1, Supplementary Material online). A phylotranscriptomic approach nested *P. streckersoni* within the family Unionidae and a close relative to *Lampsilis* and *Venustaconcha ellipsiformis* (fig. 1), aligning with previous phylogenetic studies (Smith et al. 2019, 2020). The functional annotation returned 52,407 gene models (41,065 protein-coding genes, 11,342 tRNAs). The estimated number of protein-coding genes was slightly more than other mollusk genomes of similar size (e.g., Nam et al. 2017; Wang et al. 2017), but was similar to the recent estimates in freshwater mussels (Gomes-dos-Santos et al. 2020; Rogers et al. 2020) (table 2). Coding sequences from the 41,065 predicted genes had a mean length of 375.84 amino acids and 22,568 were annotated by available protein databases. The number of annotated genes aligns with other bivalve genomes (e.g., Bai et al. 2019; Dong et al. 2020), but suggests some hypothetical proteins may be lineage specific.

In this study, I present the first high quality genome assembly for a freshwater mussel, which will be an invaluable resource for understating their biology, ecology, and evolution. Future efforts to develop a chromosome level assembly (e.g., HiC sequencing) will be useful to determine the karyotype of *P. streckersoni* and further investigate gene superfamilies. Nonetheless, this resource will facilitate genome wide association studies to screen for the genomic bases of ecologically and evolutionarily important traits such as sex determining pathways and mechanisms involved with immune evasion. Freshwater mussels also represent one of the most imperiled groups of organisms globally (Lopes-Lima et al. 2018), and this resource, along with future studies developing high-quality genome assemblies and annotations, may shed light on possible genomic characteristics that contribute to imperilment and terminal extinction.

## Supplementary Material

Supplementary data are available at *Genome Biology and Evolution* online.

## Supplementary Material

evab029_Supplementary_Data
