# Supplementary material for: A High-Quality Reference Genome for a Parasitic Bivalve with Doubly Uniparental Inheritance (Bivalvia: Unionida)
Source: Genome Biol Evol. 2021 Feb 11;13(3):evab029. doi: 10.1093/gbe/evab029 (PMC7937423; doi:10.1093/gbe/evab029)
Supplement: evab029_Supplementary_Data [file evab029_supplementary_data.zip › Supplementary_Information_v2.docx]

**Supplementary Material**

**Tables**

**Table S1.** QUAST statistics and BUSCO profiles based on the metazoan and mollusca_odb10 lineage for pooled adult and larval transcriptomes.

| Value | Adult | Glochidia |
| --- | --- | --- |
| **Transcripts** | 96,843 | 104,614 |
| **Total Length** | 105,529,389 | 113,011,961 |
| **Longest Transcript** | 36,562 | 33,017 |
| **N50** | 2,791 | 3,325 |
| **Complete Single copy** | 832 (87.2%) | 810 (84.9%) |
| **Complete Duplicated** | 87 (9.1%) | 119 (12.5%) |
| **Fragmented** | 22 (2.3%) | 17 (1.8%) |
| **Missing** | 13 (1.4%) | 8 (0.8%) |

**Table S2.** Accession numbers for taxa used in phylogenomic analyses.

| *Taxon* | Accession |
| --- | --- |
| *Amblema plicata* | SRR6793281 |
| *Aspatharia pfeifferiana* | SRR8217850 |
| *Contradens contradens* | SRR8476280 |
| *Cristaria plicata* | SRR3095781 |
| *Elliptio complanata* | SRR5136467 |
| *Elliptio crassidens* | SRR12266924 |
| *Etheria elliptica* | SRR8217851 |
| *Hyriopsis cumingii* | SRR3499637 |
| *Lamprotula leaii* | SRR6438992 |
| *Lampsilis cardium* | SRR1560282 |
| *Lampsilis siliquoidea* | SRR8356845 |
| *Margaritifera margaritifera* | SRR1560312 |
| *Megalonaias nervosa* | SRR12244633 |
| *Neotrigonia margaritacea* | SRR1560432 |
| *Potamilus streckersoni* | SRR13176627 |
| *Pyganodon grandis* | SRR910339 |
| *Scabies phaelus* | SRR8476281 |
| *Uniomerus tetralasmus* | SRR910418 |
| *Utterbackia peninsularis* | SRR6279377 |
| *Venustaconcha ellipsiformis* | SRR6279384 |

**Figure Descriptions**

**Fig. S1.** *De novo* assembled female mitochondrial genome with annotations for *Potamilus streckersoni*.

**Fig. S2.** Jellyfish and GenomeScope *k*-mer distribution and estimates of genome attributes for *Potamilus streckersoni* based on 10X Genomics reads.
