# Supplementary figures and images for: A High-Quality Reference Genome for a Parasitic Bivalve with Doubly Uniparental Inheritance (Bivalvia: Unionida)
Source: Genome Biol Evol. 2021 Feb 11;13(3):evab029. doi: 10.1093/gbe/evab029 (PMC7937423; doi:10.1093/gbe/evab029)

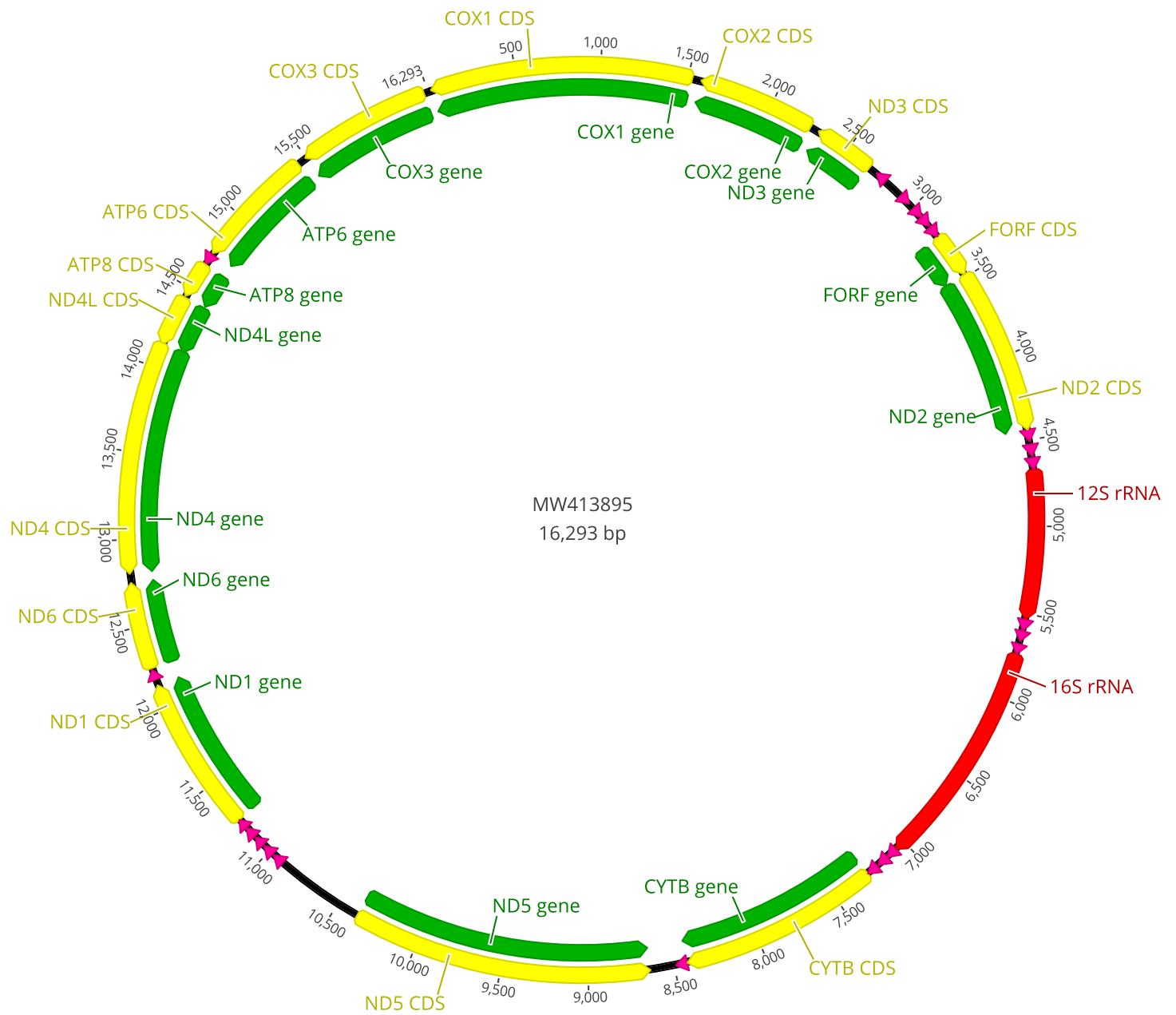

Supplement: evab029_Supplementary_Data [file evab029_supplementary_data.zip › FigureS1_Mt genome.pdf]

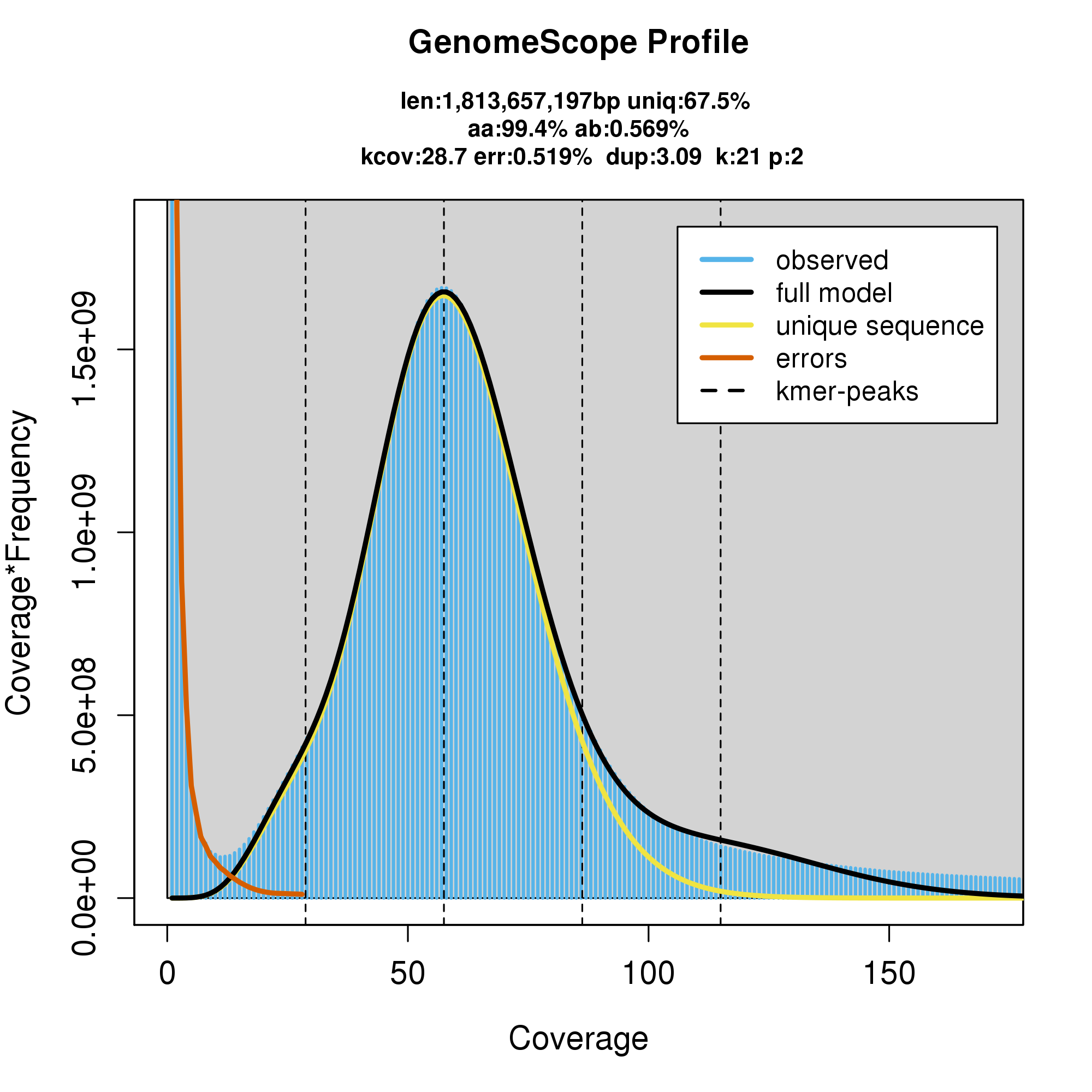

Supplement: evab029_Supplementary_Data [file evab029_supplementary_data.zip › FigureS2_GenomeScope.png]
